# Human fascioliasis infection sources, their diversity, incidence factors, analytical methods and prevention measures – CORRIGENDUM

**DOI:** 10.1017/S0031182020000256

**Published:** 2020-04

**Authors:** S. Mas-Coma, M. D. Bargues, M. A. Valero

The authors wish to apologise for an error in the Financial Support Section of the above article. The text:

Health Research Project No. PI16/00520, Subprograma Estatal de Generación de Conocimiento de la Acción Estratégica en Salud (AES), Plan Estatal de Investigación Científica y Técnica y de Innovación, ISCIII-MINECO, Madrid, Spain

should instead read:

Health Research Project No. PI16/00520, Subprograma Estatal de Generación de Conocimiento de la Acción Estratégica en Salud (AES) y Fondos FEDER, Plan Estatal de Investigación Científica y Técnica y de Innovación, ISCIII-MINECO, Madrid, Spain

## References

[ref1] Mas-Coma S, Bargues MD and Valero MA (2018) Human fascioliasis infection sources, their diversity, incidence factors, analytical methods and prevention measures. Parasitology 145, 1665–1699. 29991363 10.1017/S0031182018000914

